# Genotype and Environment Effects on Prebiotic Carbohydrate Concentrations in Kabuli Chickpea Cultivars and Breeding Lines Grown in the U.S. Pacific Northwest

**DOI:** 10.3389/fpls.2020.00112

**Published:** 2020-02-21

**Authors:** George Vandemark, Samadhi Thavarajah, Niroshan Siva, Dil Thavarajah

**Affiliations:** ^1^ Grain Legume Genetics and Physiology Research Unit, Washington State University, Pullman, WA, United States; ^2^ Plant and Environmental Sciences, Clemson University, Clemson, SC, United States; ^3^ Revelle College, University of California UC San Diego, La Jolla, CA, United States

**Keywords:** biofortification, breeding, chickpea, gut microbiome, nutrition

## Abstract

Prebiotic carbohydrates are compounds that include simple sugars, sugar alcohols, and raffinose family oligosaccharides, which are fermented by gut bacteria and can influence the species profile of the gut microbiome to reduce obesity and weight gain. Prebiotic carbohydrates are also associated with several health benefits including reduced insulin dependence and incidence of colorectal cancer. Although pulse crops such as chickpea have been important sources of nutrition for human diets for thousands of years, relatively little is known about the profiles of prebiotic carbohydrates in pulse crops. The objectives of this study were to characterize the type and concentration of seed prebiotic carbohydrates in 18 kabuli chickpea genotypes grown in 2017 and 2018 in Idaho and Washington, and partition variance components conditioning these nutritional quality traits in chickpea. Genotype effects were significant for fructose, sucrose, raffinose, and kestose. Environment effects were also significant for several carbohydrates. However, year effects were the greatest sources of variance for all carbohydrates. Concentrations of most carbohydrates were significantly greater in 2017, when there was less precipitation during the growing season coupled with greater heat stress during grain filling than in 2018. This may reflect the role of many of these carbohydrates as osmoprotectants produced in response to heat and water stress. Overall, our results suggest that a survey of more genetically diverse plant materials, such as a chickpea ‘mini-core' collection, may reveal genotypes that produce significantly greater concentrations of selected prebiotic carbohydrates and could be used to introduce desirable nutritional traits into adapted chickpea cultivars.

## Introduction

Chickpea (*Cicer arietinum* L.) was one of eight ‘founder crops' domesticated 9,000–11,000 years ago by Neolithic communities in riparian zones along the Tigris and Euphrates rivers in what is now Turkey and Syria (Lev-Yadun et al., 2000). Currently chickpea is the third most important pulse crop in terms of global production, after dry bean (*Phaseolus vulgaris* L.) and dry pea (*Pisum sativum* L.), with over 14.7 million Mt produced in 2017 (FAOSTAT, 2019). India is responsible of more than 80% of annual global production with Myanmar, Ethiopia, Turkey, and Pakistan being other major producers (FAOSTAT, 2019).

Chickpeas can be divided into two major classes, ‘kabuli' and ‘desi', based on seed characteristics. Desi chickpeas have a ‘teardrop' shape and tend to be smaller in size and have thicker and darker seed coats than kabuli chickpeas, which have a rounder shape and tend to be larger and lighter in color (Toker, 2009). Desi chickpeas are typically dehulled to remove seed coats and then split and cooked to produce dhal, or are ground to make flour, whereas kabuli chickpeas are usually cooked whole without removing seed coats and then used for salads, canned, or for making edible spreads such as hummus (Yadav et al., 2007).

The first chickpeas grown commercially in the U.S. were large, light colored kabuli chickpeas known as ‘Spanish White', which were grown in the San Joaquin Valley of southern California (Muehlbauer et al., 1982). Chickpea production began to expand in the 1980s to areas of Idaho and Washington where the predominate cropping system was dryland wheat and barley grown in rotation with lentil and pea. Commercial chickpea production in the U.S. consists almost entirely of kabuli chickpeas (Vandemark et al., 2014a). Currently chickpea is an important component of dryland production systems throughout the U.S. Pacific Northwest and Northern Plains. In 2017 more than 240,000 ha of chickpeas were harvested in the U.S. with a production value greater than $200 million (NASS, 2019). In 2017, Washington and Idaho together accounted for approximately 51% of total U.S. chickpea production, while Montana and North Dakota together accounted for approximately 43% of total production (NASS, 2019).

Biofortification, a process by which crop plants have higher concentrations of nutritional factors such as proteins, carbohydrates, or minerals, has been proposed as a way of improving human and animal nutrition (White and Broadley, 2005). Biofortification may be accomplished through management practices, the development of new cultivars with improved nutritional qualities through plant breeding, or a combination of management and genetic approaches (de Benoist et al., 2008). At least three billion people globally suffer from malnutrition caused from dietary deficiencies in iron (Fe) or zinc (Zn) (de Benoist et al., 2008; Wessells and Brown, 2012). Nutritional characterization of chickpea has largely been limited to determining seed concentrations of minerals (Bueckert et al., 2011; Ray et al., 2014; Vandemark et al., 2018) and dietary fiber (Chen et al., 2016). Non-genetic sources of variance including environment, year and their interactions have been found to have greater magnitudes of effect than genetic variance for several important minerals of global concern, including Fe, Mg, and Zn (Ray et al., 2014; Vandemark et al., 2018).

In contrast to health consequences associated with dietary deficiencies, excesses in food consumption, coupled with genetic and environmental factors, have resulted in increases in the global incidence of obesity, coronary artery disease (CAD), and diabetes. Prebiotic carbohydrates are compounds found in many food sources that have been associated with diverse health benefits (Carlson et al., 2018). The definition of ‘prebiotic' in the scientific community has evolved over more than 20 years of discussion and research and is most currently ‘A nondigestible compound that, through its metabolism by microorganisms in the gut, modulates the composition and/or activity of the gut microbiota, thus conferring a beneficial physiologic effect on the host' (Bindels et al., 2015). Prebiotic carbohydrates include the simple sugars glucose and sucrose, several sugar alcohols (SA) including sorbitol and mannitol, fructooligosaccharides (FOS) such as kestose and nystose, and raffinose family oligosaccharides (RFOs), which include raffinose, stachyose, and verbacose (Peterbauer and Richter, 2001). Prebiotic carbohydrates are fermented by gut bacteria and can influence the species profile of the gut microbiome, including increasing concentration of *Bifidobacteria* sp. that are associated with reduced obesity and weight gain (Schwiertz et al., 2010). Fermentation of prebiotic carbohydrates produces short chain fatty acids (SCFA) that are associated with several health benefits including reduced obesity and insulin dependence (Gao et al., 2009) and protection against development of colorectal cancer (Keku et al., 2015).

Significant genotype, location, and year effects have been detected for seed concentrations of several prebiotic carbohydrates in lentil (*Lens culinaris* L.), including sorbitol, mannitol, and verbacose (Johnson et al., 2013). However, the effects of genetic and non-genetic sources of variance on seed prebiotic carbohydrate concentrations have not been estimated for chickpea. Understanding these effects is essential for developing new chickpea cultivars that produce seed with higher concentrations of selected prebiotic carbohydrates across different environments. The objectives of this study were to characterize concentrations of seed prebiotic carbohydrates in 18 kabuli chickpea genotypes grown in Washington and Idaho and partition variance components conditioning these nutritional quality traits in chickpea.

## Materials and Methods

### Plant Materials and Field Trials

This study examined 18 cafe kabuli chickpea entries (**Table 1**), which included five cultivars, Billy Beans, CDC Frontier, CDC Orion, Royal, and Sierra, and 12 breeding lines. All entries were planted at two locations: Genesee, ID, (46.55° N, 116.92° W), and Pullman, WA (46.73° N, 117.18° W) in both 2017 and 2018. All seeds were treated before planting with fludioxonil (0.56 g kg^−1^, Syngenta, Greensboro, NC, USA), mefenoxam (0.38 g kg^−1^, Syngenta) and thiabendazole (1.87 g kg^−1^, Syngenta) to control fungal diseases, thiamethoxam (0.66 ml kg^−1^, Syngenta) for insect control, and molybdenum (0.35 g kg^−1^). Approximately 0.5 g *Mesorhizobium ciceri* inoculant (1 × 10^8^ CFU g^−1^; Exceed, Cambridge, MA, USA) was applied to each seed packet one day before planting. Chickpeas were planted at a density of 43 seeds m^−2^ in a 1.5 m × 6.1 m block (~430,000 seeds ha^−1^). All yield trials used a randomized complete block design with three replications. Weeds were controlled by a single post-plant/pre-emergence application of metribuzin (0.42 kg ha^−1^, Bayer Crop Science, Raleigh, NC) and linuron (1.34 kg ha^−1^, NovaSource, Phoenix, AZ, USA). All plots were exclusively rainfed and no supplemental irrigation was applied. Plots at Pullman were evaluated during the growing season for field traits including days to harvest maturity. Plots were mechanically harvested and seed yield (kg ha^−1^) determined. Hundred seed weight (HSW) was determined for each entry at Pullman by taking the average weight (g) of 100 seeds from each of three replicate plots.

**Table 1:** Mean^#^ yield, hundred seed weight (HSW) and days to mature for chickpea cultivars and breeding lines grown at Pullman, WA and Genesee, ID in both 2017 and 2018.

Entry	Pedigree	Yield (kg/ha)				HSW (g)^£^	Days to Mature^£^
		Pullman		Genesee			
		2017	2018	2017	2018		
CDC Frontier	FLIP 91-22C/ICC 14912	1163 AB	2302 AB	1980 A	3553 A	36.0 H	104 AB
CDC Orion	FLIP 95-48C/93-120-63K	1052 AB	2423 A	752 BC	3750 A	41.7 G	98 AB
Royal	HB-19/CA9783142	644 B	2175 AB	591 C	2516 A	54.8 A	106 AB
Sierra	CA188359/CA188608	809 AB	1796 C	853 BC	2757 A	49.6 BCD	103 AB
Billy Beans	Landrace	1205 AB	2264 AB	1299 ABC	3181 A	29.5 I	97 B
CA0790B0043C	HB-14/CA9783142	1141 AB	2002 AB	1126 BC	2712 A	48.3 BCDE	106 AB
CA0790B0547C	Masalla 2/CA9783153C	1350 AB	2344 AB	1039 BC	2995 A	47.8 CDEF	101 AB
CA0890B0429C	CA9990B1887C/CA9890233W	791 AB	2175 AB	1267 ABC	3481 A	52.1 ABC	106 AB
CA13900002C	PI559361/Gokge	1000 AB	2299 AB	854 BC	3067 A	45.0 EFG	101 AB
CA13900023C	CA0090B383C/Sierra	1070 AB	2023 AB	952 BC	3143 A	51.3 ABC	102 AB
CA13900046C	CA99901875W/CA0569C091	986 AB	2030 AB	1074 BC	2792 A	35.7 H	102 AB
CA13900119C	CA0469C020C/CA9890233W	897 AB	2151 AB	1309 ABC	2533 A	48.5 BCDE	105 AB
CA13900129C	CA0469C020C/CA99901604C	1435 A	2368 A	860 BC	3750 A	49.0 BCDE	110 A
CA13900139C	CA0469C020C/CA99901875W	1134 AB	2409 A	1168 BC	3769 A	45.7 DEFG	105 AB
CA13900147C	CA0469C020C/Dwelley	871 A	1937 AB	1517 AB	3105 A	51.4 ABC	105 AB
CA13900149C	CA0469C020C/Dwelley	647 B	1789 C	884 BC	2537 A	52.3 AB	108 AB
CA13900151C	CA0469C020C/Dwelley	1360 A	2316 AB	1463 AB	3322 A	43.7 FG	102 AB
CA13900162C	CA0469C020C/Sierra	1060 AB	2233 AB	1956 A	2736 A	48.9 BCDE	106 AB
Grand Mean		1034	2167	1161	3087	46.2	104

^#^ Means within a column followed by the same letter are not significantly different (Tukey's HSD, α = 0.05).

^£^Mean of yield trials conducted at Pullman, WA in 2017 and 2018.

### Prebiotic Carbohydrates

Ground seed samples (500 mg) were placed in 15-ml polypropylene conical tubes and 10 mL ddH_2_O was added to each tube, which were incubated for 1 h at 80°C (Muir et al., 2009). Samples were centrifuged at 3,000×*g* for 10 min. An aliquot (1 ml) of the supernatant was diluted with 9 ml ddH_2_O, and the diluted supernatant was filtered through a 13 mm × 0.45 µm nylon syringe filter (Fisher Scientific, Waltham, MA, USA) prior to analysis. Prebiotic carbohydrate concentrations (SA, RFO, and FOS) were measured using high performance anion exchange chromatography (HPAE) (Dionex, ICS-5000, Sunnyvale, CA, USA) as previously described (Feinberg et al., 2009; Johnson et al., 2013). SA (sorbitol and mannitol), RFO (raffinose, stachyose, and verbascose), and FOS (kestose) were identified and quantified using pure standards (> 99%), and concentrations were detected within a linear range of 3 to 1,000 μg g^−1^ with a minimum detection limit of 0.2 μg g^−1^. A lab reference (CDC Redberry lentil) was used to ensure the accuracy and reproducibility of detection. The peak areas of the external reference, glucose (100 ppm), SA (3–1,000 ppm), RFO (3–1,000 ppm), and FOS (3–1,000 ppm) were routinely analyzed for method consistency and detector sensitivity, with an error of less than 5%.

### Resistant Starch

RS concentrations were determined as previously described (McCleary and Monaghan, 2002) using a commercial assay (Megazyme, 2012). Ground samples (500 mg) were incubated with 4 ml of 100 mM sodium malate (pH 6) containing α-amylase (10 mg ml^−1^) and amyloglucosidase (3 U ml^−1^) for 16 h in a water bath (37°C) with 200 strokes/min vertical shaking (Orbit shaker bath, Lab Line Instruments Inc., Melrose Park, IL, USA). After incubation, 4 ml of 95% ethanol were added, and the samples were centrifuged at 1,500×*g* for 10 min at room temperature. The pellets were re-suspended with 6 ml of ethanol (50% v/v), centrifuged, and decanted. The resuspension and centrifugation processes were done twice. Supernatants from the three centrifugations were pooled and brought to a volume of 100 ml in ddH_2_O. The pellets were dissolved in 2 ml of potassium hydroxide (2 M) in an ice bath (~0°C) while stirring with a magnetic stirrer for 20 min. The suspensions were diluted with 8 ml of sodium acetate buffer (1.2 M, pH 3.8), with 0.1 ml of 3,300 U ml^−1^ amyloglucosidase then immediately added followed by incubation at 50°C for 30 min. The suspension was then centrifuged at 1,500×*g* for 10 min at room temperature. Aliquots (0.1 ml) of both the supernatant containing the RS fractions and the diluted washings containing the soluble starch (SS) fractions were transferred separately to 10-ml glass tubes. A reagent blank was prepared using 0.1 ml sodium acetate buffer (pH 4.5). An aliquot (3 ml) of GOPOD reagent was added to each tube, which were incubated in a water bath at 50°C for 20 min. Absorption was measured using a spectrophotometer (Genesys 20, Thermo Scientific, NC, USA) at 510 nm. Starch fractions were calculated as follows:

$$\text{RS}=\frac{\text{X}\;\times \;(\text{Ab}{\text{s}}_{\text{sample}})}{\left(\text{Ab}{\text{s}}_{\text{glucose}}\times \;{\text{W}}_{\text{sample}}\right)},$$

$$\text{SS}=\frac{\text{Y}\times (\text{Ab}{\text{s}}_{\text{sample}})}{(\text{Ab}{\text{s}}_{\text{glucose}}\times {\text{W}}_{\text{sample}}},$$

where Abs_sample_ and Abs_glucose_ are the absorbance value of sample and glucose corrected against reagent blank, respectively; W_sample_ is the moisture corrected weight of sample; and X and Y are the dilutions factors for RS and SS, respectively. Regular corn starch (RS concentration 1.0 ± 0.1% (w/w)) was used to verify the data, and batches were checked regularly to ensure an analytical error of less than 10%.

### Chemicals

Solvents and standards used for high performance anion exchange chromatography (HPAE) and enzymatic assays were purchased from Fisher Scientific (Asheville, NC, USA), Sigma-Aldrich (St. Louis, MO, USA), and VWR International (Satellite Blvd, Suwanee, GA, USA). Distilled and deionized water (ddH_2_O; NANO-pure Diamond, Barnstead, IA, USA) was used in these analyses.

### Statistical Analysis

Entries (genotypes) were considered fixed factors and locations (environments), replications (blocks) within locations, and years were considered random factors. Combined ANOVA was conducted across both locations and years to detect effects of genotypes, environments, and their interactions. Entry means were compared between all pairs using Tukey's HSD test (*α* = 0.05). Pairwise correlations were determined between seed carbohydrate concentrations and yield from data combined across both locations and years, and correlations were also determined between carbohydrate concentrations, HSW and days to mature for data obtained at Pullman, WA in 2017 and 2018. All statistical analyses were performed with JMP software (SAS, Cary, NC, USA).

## Results

### Chickpea Seed Carbohydrate Concentrations

Mean squares of combined analysis of variance for chickpea seed carbohydrate concentrations are presented in **Table 2**. Genotype effects were significant for fructose, sucrose, raffinose, and kestose. Genotype effects were greatest for the simple sugars fructose and sucrose. Environment effects were also significant for several carbohydrates including sorbitol, glucose, fructose, kestose, and soluble starch. Environment effects were greatest for fructose, soluble starch, and glucose. Year effects were significant for all carbohydrates. Year effects were the greatest sources of variance for all carbohydrates. A significant genotype × environment effect was only observed for fructose. Significant genotype × year effects were observed for fructose and raffinose, however, the magnitudes of these effects were minor in comparison with year effects. Environment × year effects were significant for all carbohydrates except sucrose, verbacose, and soluble starch. The greatest interaction effect for all carbohydrates was the environment × year effect. A significant genotype × environment × year effect was only observed for fructose.

**Table 2:** Mean squares of combined ANOVA, and coefficient of variation (CV) for concentrations of prebiotic carbohydrates in chickpea cultivars and breeding lines grown in Idaho and Washington^#^.

Prebiotic Carbohydrate	Genotype (G)	Environment (E)	Year (Y)	G x E	G x Y	E x Y	G x E x Y	CV (%)
Sorbitol	26,215	102,540*	11,511,773***	17,334	18,696	280,608***	17,710	18.6
Mannitol	146	843*	27,467***	36	128	905*	52	71.1
Glucose	52	2,295***	7,587***	103	62	1,820***	88	34.2
Fructose	25***	471***	354***	12***	19***	191***	14***	78.8
Sucrose	543,676***	43,134	24,978,312***	118,945	75,914	215,543	108,939	17.5
Stachyose	49,531	100,736	27,014,050***	72,468	53,655	1,864,656***	67,584	18.2
Raffinose	22,926**	5,091	873,905***	9,544	15,955*	329,788***	7,615	19.4
Verbascose	12,403	8,481	17,172,870***	8,683	10,780	15,715	8,169	28.6
Kestose	258*	1,328*	17,612***	171	177	733*	181	43.9
Res. starch^£^	1.3	15.8*		2.5				29.7
Sol. starch	92	763***	2,014***	74	55	421	63	19.2

# Study included 18 kabuli genotypes evaluated at two environments (Pullman, WA and Genesee, ID) in 2017 and 2018.

^£^ Resistant starch concentrations were only determined for samples harvested at Pullman and Genesee in 2017.

* Significant at P < 0.05.

** Significant at P < 0.001.

*** Significant at P < 0.0001.

The most abundant carbohydrate in chickpea seed was sucrose, which on average constituted greater than 1.6% of total seed weight, followed by stachyose and sorbitol (**Table 3**). Sucrose represented greater than 95% of total simple sugars (sucrose + fructose + glucose). Stachyose represented greater than 50% of total RFO (stachyose + raffinose + verbacose), which was the most abundant class of prebiotic carbohydrates. The least abundant carbohydrates in chickpea seed were fructose and mannitol. Concentrations of glucose and kestose were similar in chickpea seeds. Significant differences between means of chickpea entries were detected only for seed concentrations of sucrose. CA13900023C had a significantly higher sucrose concentration than CA13900046C, but no other significant differences were detected. Soluble starch on average constituted 41% of total seed weight and was approximately 10× more abundant than resistant starch.

**Table 3:** Mean^#^ concentrations of prebiotic carbohydrates for chickpea cultivars and breeding lines grown at Pullman, WA and Genesee, ID in both 2017 and 2018.

Entry	Sorbitol mg/100 g	Mannitol mg/100 g	Glucose mg/100 g	Fructose mg/100 g	Sucrose mg/100 g	Stachyose mg/100 g	Raffinose mg/100 g	Verbacose mg/100 g	Kestose mg/100 g	Soluble Starch g/100 g
Billy Beans	708 A	11.0 A	28.0 A	0.82 A	1,378 AB	1,235 A	406 A	340 A	25.2 A	38.9 A
CA0790B0043C	710 A	12.2 A	28.6 A	4.82 A	1,911 AB	1,228 A	514 A	355 A	30.3 A	41.5 A
CA0790B0547C	606 A	15.3 A	27.7 A	3.36 A	1,921 AB	1,175 A	534 A	310 A	24.9 A	39.8 A
CA0890B0429C	660 A	12.8 A	27.3 A	4.53 A	1,758 AB	1,108 A	408 A	246 A	17.2 A	39.1 A
CA13900002C	678 A	10.4 A	29.5 A	2.38 A	1,610 AB	1,239 A	442 A	340 A	20.9 A	41.3 A
CA13900023C	674 A	13.4 A	31.5 A	4.90 A	2,034 A	1,307 A	518 A	377 A	36.3 A	48.1 A
CA13900046C	747 A	7.3 A	30.6 A	0.91 A	1,337 B	1,241 A	415 A	331 A	26.0 A	39.2 C
CA13900119C	799 A	12.5 A	30.1 A	1.44 A	1,656 AB	1,167 A	447 A	333 A	26.6 A	42.9 A
CA13900129C	757 A	12.0 A	29.0 A	1.51 A	1,881 AB	1,223 A	484 A	359 A	27.0 A	47.4 A
CA13900139C	710 A	16.5 A	31.5 A	1.44 A	1,766 AB	1,312 A	495 A	376 A	27.4 A	40.6 A
CA13900147C	765 A	9.3 A	29.9 A	1.14 A	1,479 AB	1,243 A	401 A	330 A	30.5 A	38.8 A
CA13900149C	728 A	8.0 A	30.2 A	1.30 A	1,566 AB	1,344 A	446 A	371 A	29.3 A	40.7 A
CA13900151C	701 A	15.4 A	30.1 A	1.22 A	1,485 AB	1,217 A	437 A	328 A	27.7 A	38.9 A
CA13900162C	670 A	15.0 A	33.3 A	2.39 A	1,858 AB	1,147 A	447 A	332 A	27.9 A	41.9 A
CDC Frontier	670 A	11.9 A	24.8 A	1.07 A	1,391 AB	1,140 A	385 A	281 A	19.7 A	38.7 A
CDC Orion	672 A	15.7 A	32.6 A	2.58 A	1,802 AB	1,321 A	476 A	361 A	23.0 A	41.1 A
Royal	672 A	20.2 A	31.2 A	1.17 A	1,884 AB	1,284 A	499 A	343 A	30.6 A	39.5 A
Sierra	649 A	19.5 A	27.0 A	4.29 A	1,777 AB	1,169 A	438 A	321 A	20.9 A	39.9 A
Grand Mean	698	13.3	29.6	2.31	1696	1,228	455	335	26.2	41.0

^#^ Means within a column followed by the same letter are not significantly different (Tukey's HSD, α = 0.05).

Mean concentrations of carbohydrates across locations and years are presented in **Table 4**. For the majority of carbohydrates, including sorbitol, mannitol, glucose, sucrose, stachyose, raffinose, verbacose, and kestose, mean concentrations at both locations in 2017 were significantly greater than both locations in 2018. Significant differences in mean concentrations of carbohydrates between Pullman-2017 and Genesee-2017 were only observed for mannitol and raffinose. Significant differences in mean concentrations between Pullman-2018 and Genesee-2018 were observed for several carbohydrates including sorbitol, glucose, fructose, raffinose, kestose, and soluble starch.

**Table 4:** Mean^#^ concentrations by location and year of prebiotic carbohydrates for chickpea cultivars and breeding lines grown at Pullman, WA and Genesee, ID in both 2017 and 2018.

Location-Year	Sorbitol mg/100 g	Mannitol mg/100 g	Glucose mg/100 g	Fructose mg/100 g	Sucrose mg/100 g	Stachyose mg/100 g	Raffinose mg/100 g	Verbacose mg/100 g	Kestose mg/100 g	Soluble Starch g/100 g
Genesee 2017	917 A	20.7 B	35.1 A	1.26 B	2,057 A	1,659 A	554 A	635 A	36.0 A	43.5 A
Pullman 2017	945 A	28.6 A	35.9 A	1.55 B	2,016 A	1,509 A	484 B	603 A	34.6 A	44.5 A
Genesee 2018	523 B	1.82 C	17.3 C	1.29 B	1,308 B	1,308 B	335 D	46.0 B	21.5 B	34.7 B
Pullman 2018	406 C	1.77 C	29.8 B	6.03 A	1,393 B	1,393 B	435 C	50.5 B	12.6 C	41.3 A

^#^ Means within a column followed by the same letter are not significantly different (Tukey's HSD, α = 0.05).

### Correlations Between Carbohydrate Concentrations, Yield, HSW, and Days to Mature

Significant correlations (P <0.05) between carbohydrate concentrations were observed for the majority of pairwise combinations and only correlations with r ≥0.80 will be noted. The highest positive correlations between carbohydrate concentrations were observed between verbacose and sorbitol (r = 0.93), verbacose and stachyose (r = 0.92), stachyose and sorbitol (r = 0.88), stachyose and sucrose (r = 0.85), and verbacose and sucrose (r = 0.82).

Correlations between seed carbohydrate concentrations and agronomic traits tended to be less than those observed between different carbohydrate concentrations. Correlations between carbohydrate concentrations and HSW or days to flower had relatively low magnitude (r <0.40) or not significant. Correlations between carbohydrate concentrations and days to mature tended to positive for most carbohydrates and were highest for sorbitol (r = 0.67) and verbacose (r = 0.65). However, significant negative correlations of appreciable magnitude were observed between several carbohydrate concentrations and plot yield. The highest negative correlations with yield were observed for the RFOs verbacose (r = −0.80) and stachyose (r = −0.77), followed by simple sugars sorbitol (r = −0.66) and mannitol (r = −0.65).

## Discussion

Significant genotype effects were detected for several prebiotic carbohydrates (**Table 2**). However, non-genetic sources of variance including year effects and environment × year interaction effects were the greatest sources of variance for all carbohydrates (**Table 2**). These results suggest that only limited gains may be made in these traits using adapted parental materials. Minor genotype effects, or in many cases a lack of significant genotype effects are likely due in part to the relatively narrow genetic base present in the examined chickpea cultivars and breeding lines (**Table 1**). Three breeding lines are full-sibs derived from CA0469C020C/Dwelley and seven breeding lines share as a parent CA0469C020C, which has resistance to Ascochyta blight and is a full-sib line to CA0469C025C, a germplasm with improved disease resistance and high yield (Vandemark et al., 2014b).

Significant environment effects were detected for several prebiotic carbohydrates (**Table 2**). Although only two environments were examined, these results suggest improved understanding of factors contributing to environmental and management sources of variance may promote reliable production of more nutritious chickpeas. The absence of significant genotype × environment interaction effects observed in this study for all carbohydrates except fructose (**Table 2**) can likely be attributed to limited genetic variation between plant materials and similarities between the two test locations.

Year effects were the greatest source of variance for all carbohydrate concentrations (**Table 2**). For the majority of carbohydrates, mean concentrations in 2017 were significantly greater than in 2018 (**Table 4**). Monthly average temperatures and total monthly precipitation are presented in **Table 5** for Pullman, WA and Genesee, ID during 2017 and 2018. Average temperatures early in the growing season (April and May) were warmer in 2018 than 2017 at Pullman and Genesee. However, average temperatures later in the growing season (July and August) were cooler in 2018 than 2017 at both locations. Both locations received more precipitation early in the growing season (April and May) in 2018 than 2017. These data suggest that the higher concentrations of many carbohydrates observed in 2017 may be the result of lower precipitation during the growing season coupled with greater heat stress later in the season (July and August) during grain filling. This may reflect the role of many of these compounds as osmoprotectants produced in response to heat and water stress.

**Table 5:** Average monthly temperature and precipitation during growing season in Pullman^#^, WA and Genesee^£^, ID in 2017 and 2018.

	Average Temperature (°C)				Total Precipitation (mm)			
	Pullman 2017	Pullman 2018	Genesee 2017	Genesee 2018	Pullman 2017	Pullman 2018	Genesee 2017	Genesee 2018
April	7.4	7.9	6.6	6.9	36.6	45.5	76.7	97.0
May	12.2	14.7	11.7	14.2	39.6	47.0	57.2	67.3
June	16.1	15.1	15.6	14.4	20.8	22.9	38.6	40.1
July	20.7	19.7	20.5	19.4	0.5	0	0.3	1.0
August	20.4	19.1	20.4	19.1	1.0	6.9	3.0	18.0

^#^ Data from Washington State University AgWeatherNet (https://weather.wsu.edu).

^£^ Data from U.S. National Center for Climate Information (https://www.ncdc.noaa.gov).

Total RFO content in chickpea seed averaged 2.0% of dry weight, which is consistent with reports for other seeds ranging from 2 to 10% (Peterbauer and Richter, 2001). The most abundant RFO in chickpea seed was stachyose (**Table 2**). This is consistent with previous reports for other legume seeds, including dry bean (*P. vulgaris* L.) (McPhee et al., 2002) and soybean (*Glycine max* L.) (Kumar et al., 2010) for which stachyose was more abundant than raffinose.

A positive correlation with r >0.80 was observed between seed concentrations of verbacose and stachyose. This likely reflects their shared RFO biosynthetic pathway in seeds, in which galactosylation of raffinose leads to production of stachyose, to which an additional galactosyl residue is transferred to produce verbacose (Peterbauer and Richter, 2001). Similarly high correlations were also observed between these two RFOs, sucrose, and sorbitol. The high correlations between sucrose, stachyose, and verbacose can also be explained by the role of sucrose as the first galactosyl residue acceptor in the RFO biosynthetic pathway. High correlations between sorbitol, stachyose and verbacose likely reflect that along with sucrose, SA such as sorbitol are primary products of photosynthesis and a major source of translocated carbohydrate to seed (Slewinski and Braun, 2010).

Only minor or non-significant correlations were observed between seed carbohydrate concentrations and seed size (HSW). However, high negative correlations were observed between yield and concentrations of RFOs verbacose and stachyose, and between yield and SAs sorbitol and mannitol. Although RFOs primarily function to store carbon in seeds, they are also known to accumulate in response to abiotic stress factors including heat (Panikulangara et al., 2004) and drought (Downie et al., 2003). Sorbitol has been shown to accumulate in several plant species in response to various abiotic factors including osmotic (Pommerrenig et al., 2007) and drought stress (Li et al., 2012). Similarly, accumulation of mannitol has been shown to increase tolerance to drought stress in several plant species (Patonnier et al., 1999; Abebe et al., 2003). Climatic conditions that contributed to lower yields in 2017, including higher temperatures during grain filling and lower precipitation (**Table 5**), also likely resulted in higher seed concentrations of carbohydrates associated with drought and heat stress.

Identifying sources of genetic variation in chickpea for seed concentrations of prebiotic carbohydrates and understanding the magnitude of genotype, environment, and their interaction effects on these traits are important for accelerating progress in breeding more nutritious chickpea cultivars. In this study non-genetic effects contributed more than genetic effects to total variation in carbohydrate concentrations, suggesting there is very limited genetic variation for these traits in the elite chickpea breeding lines and cultivars examined in this study. However, a survey of more genetically diverse plant materials, such as a chickpea ‘mini-core' collection (Upadhyaya and Ortiz, 2001) may reveal chickpea genotypes that produce exceptionally high concentrations of selected prebiotic carbohydrates and could be used to introduce desirable nutritional traits into adapted chickpea cultivars.

## Data Availability Statement

The datasets generated for this study are available on request to the corresponding author.

## Author Contributions

GV and DT conceived this work. GV planned and carried out field experiments including data collection, harvesting and cleaning seed samples. DT directed laboratory work to determine prebiotic carbohydrate profiles, maintained equipment for high performance anion exchange chromatography (HPAE), and analyzed data. ST and NS performed laboratory work and collected data. GV performed statistical analysis. GV and DT drafted the manuscript. All authors read and approved the manuscript.

## Funding

This work was funded by a U.S. Department of Agriculture, Agricultural Research Service Pulse Crop Health Initiative competitive grant (‘Improving the nutritional value of chickpeas’).

## Conflict of Interest

The authors declare that the research was conducted in the absence of any commercial or financial relationships that could be construed as a potential conflict of interest.
